# Microalgal Cultures for the Bioremediation of Urban Wastewaters in the Presence of Siloxanes

**DOI:** 10.3390/ijerph19052634

**Published:** 2022-02-24

**Authors:** Eva M. Salgado, Ana L. Gonçalves, Francisco Sánchez-Soberón, Nuno Ratola, José C. M. Pires

**Affiliations:** 1 LEPABE—Laboratory for Process Engineering, Environment, Biotechnology and Energy, Faculty of Engineering, University of Porto, Rua Dr. Roberto Frias, 4200-465 Porto, Portugal; up201606419@edu.fe.up.pt (E.M.S.); algoncalves@fe.up.pt (A.L.G.); fssoberon@fe.up.pt (F.S.-S.); nrneto@fe.up.pt (N.R.); 2 ALiCE—Associate Laboratory in Chemical Engineering, Faculty of Engineering, University of Porto, Rua Dr. Roberto Frias, 4200-465 Porto, Portugal

**Keywords:** contaminants of emerging concern, microalgae, nutrients removal, siloxanes, urban effluents, wastewater bioremediation

## Abstract

Microalgae are widely used in the bioremediation of wastewaters due to their efficient removal of pollutants such as nitrogen, phosphorus, and contaminants of emerging concern (CECs). Siloxanes are CECs that reach wastewater treatment plants (WWTPs), leading to the production of biogas enriched with these compounds, associated with the breakdown of cogeneration equipment. The biological removal of siloxanes from wastewaters could be a sustainable alternative to the costly existing technologies, but no investigation has been performed using microalgal cultures for this purpose. This study evaluated the ability of *Chlorella vulgaris* to bioremediate primary (PE) and secondary (SE) urban effluents and remove volatile methylsiloxanes (VMSs). *C. vulgaris* grew successfully in both effluents, and approximately 86% of nitrogen and 80% of phosphorus were efficiently removed from the PE, while 52% of nitrogen and 87% of phosphorus were removed from the SE, and the presence of VMSs does not seem to have a negative influence on nutrient removal. Three out of the seven of the analysed VMSs were detected in the microalgal biomass at the end of the PE assay. However, dodecamethylcyclohexasiloxane (D6) was the one that accumulated to a greater extent, since 48% of the initial mass of D6 was detected in the biomass samples. D6 is one of the most lipophilic VMSs, which might contribute to the higher adsorption onto the surface of microalgae. Overall, the results indicate *C. vulgaris*’ potential to remove specific VMSs from effluents.

## 1. Introduction

Microalgae can be defined as a diverse group of photosynthetic microorganisms comprising eukaryotic microalgae and prokaryotic cyanobacteria [1]. These microorganisms are used in various applications, but one of the most remarkable is in wastewater bioremediation, particularly in secondary and tertiary treatments of wastewater treatment plants (WWTPs). Microalgae-based treatment systems can be more efficient than traditional systems, requiring lower capital investment and operation costs and providing natural disinfection [2]. *Chlorella vulgaris* is one of the most used microalgal species for this application, due to its high adaptability to different environmental conditions and resistance to heavy metals and other potentially inhibitory compounds [3,4]. Znad et al. [5] showed that this microalga could efficiently remove nitrogen and phosphorus from both primary- and secondary-treated municipal effluents at different dilutions, with removal efficiencies over 80%. Furthermore, their specific growth rate increased with increasing effluent fractions, showing that culturing microalgae in municipal effluents can be a cost-effective approach for wastewater purification. Zhou et al. [6] demonstrated that *C. vulgaris* could easily grow in filtered municipal raw wastewater (influent) and not only provide very high nutrient removal efficiencies (i.e., 92% for total nitrogen (TN) and 82% for total phosphorus (TP)), but also remove metals and organic contaminants. *C. vulgaris* has also been demonstrated to adapt to highly concentrated municipal wastewater streams generated by the dewatering of sludge from primary- and secondary-settling (centrate), as proposed by Zhou et al. [7]. Besides municipal, urban or domestic wastewaters, several studies have shown the great bioremediation potential of this microalga on nutrients’ removal from hydroponic greenhouse cultivation wastewaters, piggery wastewaters, textile wastewaters, paper industry effluents, palm oil mill effluents, riboflavin manufacturing wastewaters, and fertiliser industrial wastewaters, among others [8,9,10,11,12].

In addition to inorganic pollutants such as nitrogen, phosphorus and metals, wastewaters (particularly domestic) can contain organic pollutants, such as endocrine-disrupting chemicals (EDCs), pharmaceuticals and personal care products (PPCPs), pesticides, and siloxanes, which nowadays are considered contaminants of emerging concern, CECs [6,13]. Siloxanes can be defined as chemically synthesised polymeric organic silicon molecules that consist of a backbone of alternating Si-O units with organic sidechains attached to each silicon atom [14]. Due to their antimicrobial properties, hydrophobicity and biocompatibility, siloxanes are used in various industrial applications, such as cosmetics, personal care products (PCPs), the food industry, and medicine, among others [15]. Within the siloxane family, volatile methylsiloxanes (VMSs) are oligomeric alkylsiloxanes with: (i) low molecular weight; (ii) low water solubility that decreases with the increase in their chain length; (iii) relatively high vapour pressures; and (iv) low boiling points, therefore having a strong tendency to volatilise from aqueous solutions. These compounds have high partition coefficients between octanol and water (*K_OW_*) and between organic carbon and water (*K_OC_*), which can indicate high adsorption of compounds onto the solid phase or the surface of microorganisms [16,17,18,19]. Cyclic volatile methylsiloxanes (cVMSs) (e.g., octamethylcyclotetrasiloxane (D4), decamethylcyclopentasiloxane (D5), and dodecamethylcyclohexasiloxane (D6)) are the subject of considerable scientific interest and recent regulatory discussion and implementation of restrictions to their use in PCPs [20,21], since they have been widely and increasingly produced and used, leading to their widespread detection in water, air, sediments and biota [22,23].

Due to the daily use of products containing siloxanes and their chemical derivatives, these pollutants are frequently found in WWTPs, predominantly D4 and D5, while the remaining siloxanes appear in lower amounts [24,25]. In WWTPs, their accumulation in sludge is favoured, which leads to the production of biogas enriched with these compounds [26]. When combusted to generate energy, siloxanes are oxidised into microcrystalline silicon dioxide that deposits on cogenerator parts [27]. These oxides are highly abrasive, wearing down the equipment, changing the combustion chamber in geometry and clogging lines, which could cause accidental explosions, decrease the system’s useful lifetime, and increase operational and maintenance costs [24,25]. Current technologies to remove siloxanes from biogas have numerous limitations, such as their high implementation and operational costs. Biological removal of siloxanes from wastewaters instead of biogas could be an economical and environmentally friendly alternative, contributing to minimising soil and aquatic ecosystems contamination. Even though siloxane removal and degradation studies have been conducted with bacteria [28,29,30], no investigation has been performed with microalgae.

*C. vulgaris* has great potential to remove and even degrade several CECs from the culture medium. For instance, Baglieri et al. [8] proposed that this microalga degraded pesticides such as fenhexamid and metalaxyl, present in the culture medium, while pyrimethanil was absorbed onto microalgal cells. Furthermore, a study conducted by Gojkovic et al. [31], aiming to remove several active pharmaceutical ingredients (APIs) from the culture medium using *C. vulgaris*, showed significant removal efficiencies of up to 98% and 100% for diphenhydramine and memantine, respectively. Many other compounds were removed from the culture medium, and some were also shown to accumulate in the algal biomass, such as mirtazapine and oxazepam. In general, lipophilic pharmaceuticals were efficiently removed and accumulated in the algal biomass to a greater extent than polar APIs. With this in mind, it appears plausible that this microalgal species might be able to remove lipophilic compounds such as siloxanes from wastewaters. Accordingly, this study aimed to evaluate the microalgal bioremediation of primary and secondary treated urban wastewaters using *C. vulgaris* and, for the first time, determine if they can also remove VMSs. Since microalgae can be applied during either secondary or tertiary treatments, it is important to evaluate nutrient removal in both effluents, taking the opportunity to monitor VMSs concentration in different matrices over time. Moreover, it is important to use wastewaters with different initial VMSs concentrations to assess their behaviour. Primary effluents typically have higher concentrations compared to secondary effluents, since during the secondary treatment, the aeration process can lead to the volatilisation of a great percentage of these compounds [24].

## 2. Materials and Methods

### 2.1. Chemicals and Materials

#### 2.1.1. Urban Effluents

**Figure 1:**
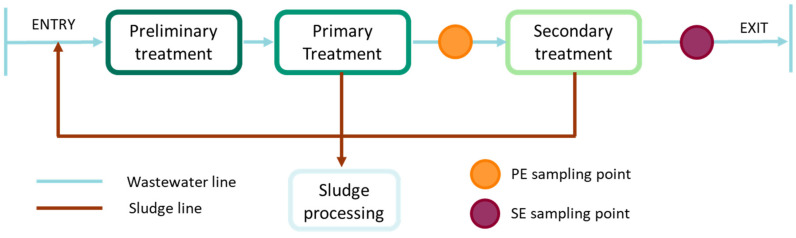
Scheme of the treatment process and effluent sampling points in the WWTP selected for this study.

The effluents used in this study were collected from a Portuguese WWTP with over 100,000 population equivalents. The wastewater treatment process in this WWTP, presented in Figure 1, consists of: (i) a preliminary treatment, in which solids, coarse elements, sand and grease are removed by filtration through bar screens and physical separation in aerated grit-grease removers; (ii) a primary treatment, where primary sludge is separated from grease and oils in large sedimentation tanks; (iii) a secondary or biological treatment (aerobic digestion), in which dissolved and suspended organic matter is significantly reduced in aeration tanks or biological reactors. The sludge collected from primary and secondary treatments undergoes several processing steps, such as thickening and anaerobic digestion, leading to biogas production. From this WWTP, two effluent types were collected: one after the primary treatment step (primary effluent, PE) and another after the secondary treatment step (secondary effluent, SE). Effluent samples were stored at −20 °C until the effluent characterisation was performed.

#### 2.1.2. Microalgae and Culture Medium

The microalga used in this project, *C. vulgaris* CCAP 211/11B, was obtained from the Culture Collection of Algae and Protozoa (CCAP, Oban, UK). Stock solutions were prepared in 100 mL Erlenmeyer flasks, with a working volume of 50 mL, using the modified OECD (Organisation for Economic Cooperation and Development) test medium, with the following composition (per litre): 250 mg NaNO_3_; 18 mg CaCl_2_·2H_2_O; 12 mg MgCl_2_·6H_2_O; 45 mg KH_2_PO_4_; 15 mg MgSO_4_·7H_2_O; 185 µg H_3_BO_3_; 0.08 mg FeCl_3_·6H_2_O; 0.1 mg Na_2_EDTA·2H_2_O; 415 µg MnCl_2_·4H_2_O; 3 µg ZnCl_2_; 1.5 µg CoCl_2_·6H_2_O; 7 µg Na_2_MoO_4_·2H_2_O; 0.01 µg CuCl_2_·2H_2_O; and 500 mg NaHCO_3_. The Erlenmeyer flasks were maintained at room temperature under a continuous light supply of approximately 6.50 µmol m^−2^ s^−1^. Agitation was promoted through an Unimax 1010 orbital shaker (Heidolph, Schwabach, Germany), set at 100 rotations per minute (rpm). After 30 days of cultivation, the stock solutions were transferred to five 500 mL glass bottles that were maintained under the same light and temperature conditions. After 15 days, cells grown in the 500 mL bottles were harvested through centrifugation at 12,000 rpm (22,114× *g*), 20 °C, for 10 min in an Avanti J-25 centrifuge (Beckman, Brea, CA, USA).

#### 2.1.3. Chemicals for Siloxane Analyses

Seven VMSs, three linear (octamethyltrisiloxane (L3), decamethyltetrasiloxane (L4) and dodecamethylpentasiloxane (L5)), and four cyclic (hexamethylcyclotrisiloxane (D3), D4, D5 and D6), were analysed in the present study. Tetrakis (trimethylsilyloxy)silane (M4Q) was used as the internal standard (IS). Individual VMSs standards and M4Q with a purity level higher than 97% were purchased from Sigma-Aldrich (St. Louis, MO, USA). Individual 1 g L^−1^ stock solutions of these standards in *n*-hexane and then a final 5 mg L^−1^ stock mixture were prepared to be used in the construction of the calibration curves. The 5 mg L^−1^ M4Q standards used in the sample extraction procedures were prepared in analytical grade *n*-hexane or acetone, obtained from VWR (Fontenay-sous-Bois, France), and protected from light in amber glass vials. All preparations were kept in the dark at −20 °C until use. Specifically for the QuEChERS procedure, the following reagents were used: ethyl acetate and dichloromethane from VWR (Fontenay-sous-Bois, France), anhydrous magnesium sulphate from Panreac AppliChem (Barcelona, Spain), primary and secondary amine (PSA) and octadecylsilane (C18) from Supelco (Bellefonte, PA, USA). Nitrogen for sample evaporation with a purity of 99.9999% was provided by Air Liquide (Maia, Portugal).

### 2.2. Effluent Characterisation

Before microalgal cultivation, the effluents were allowed to settle overnight and then filtered through GF/A-grade glass microfibre filters (Whatman, Maidstone, UK), using a vacuum filtration system. The physicochemical characteristics of both raw and filtered PE and SE are presented in Table 1. The methodology used to characterise these effluents was as follows: (i) pH and conductivity were determined using a Consort’s C6010 electrochemical analyser (Brussels, Belgium); (ii) colour was determined in Hazen units, as described in the Portuguese Standard NP-627:1972 [32]; (iii) turbidity was measured using a Hanna Instruments HI88703 turbidimeter (Rhode Island, USA); (iv) chemical oxygen demand (COD), TP, total solids (TS) and total volatile solids (TVS) were determined according to the Standard Methods for the Examination of Water and Wastewater, through the 5220-D, 4500-P E, 2540 B and 2540 E tests, respectively [33]; (v) total dissolved carbon (TDC), dissolved organic carbon (DOC), dissolved inorganic carbon (DIC) and total nitrogen (TN) were determined by filtrating the effluent using 0.22-µm cellulose acetate membranes (Orange Scientific, Braine-l’Alleud, Belgium) and analysing the filtrate in an organic carbon analyser (TOC-VCSN, Shimadzu); (vi) nitrate-nitrogen (NO_3_-N) concentration was determined using the United States Environmental Protection Agency (USEPA)-approved Brucine Colorimetric Method [34]; and (vii) phosphate-phosphorus (PO_4_-P) was quantified using the ascorbic acid colorimetric method, as described by Lee et al. [35]. The calibration curve data for each of these determinations are presented in Table S1 of the Supplementary Material (SM). VMSs levels in the effluents were determined as described in Section 2.6. The raw PE revealed much higher colour, turbidity, DOC, COD, TS and TVS values than the raw SE, in which the organic matter was in its oxidised form due to the biological oxidation step. In the PE, even though P was in the oxidised form, N was still in the reduced form, as it did not go through oxidation. For this reason, NO_3_-N was not detected in this effluent. Nitrogen and phosphorus in the SE were mainly in the oxidised form, which explains the similarity between TN and TP values with the NO_3_-N and PO_4_-P levels, respectively.

### 2.3. Experimental Setup

The experiments regarding microalgal growth in previously filtered PE and SE were conducted in batch mode using 5 L bottles as a cultivation system. The following experimental conditions were tested: (i) a positive control assay (C+) containing the *C. vulgaris* inoculum with 4.5 L of the modified OECD test medium; (ii) a negative control assay (C-PE) containing only 4.5 L of filtered PE; (iii) an assay with *C. vulgaris* inoculum and 4.5 L of filtered PE (PE); (iv) a negative control assay (C-SE) containing only 4.5 L of filtered SE; and (v) an assay with *C. vulgaris* inoculum and 4.5 L of filtered SE (SE). Two independent experiments were performed for each tested condition. In the C-PE assay, one experiment was performed without light exposure by wrapping the bottle in aluminium paper, thus eliminating the possibility of siloxanes removal by photodegradation.

**Figure 2:**
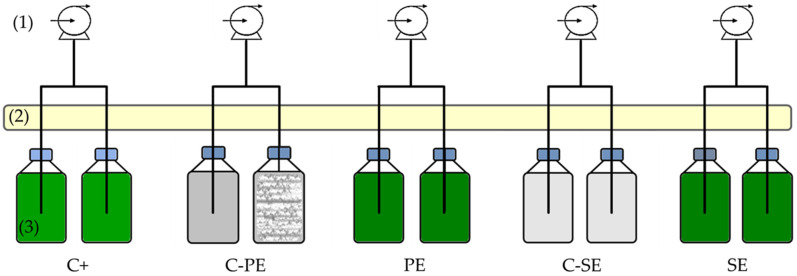
Schematic representation of the experimental setup: (**1**) air pumps to provide atmospheric air and agitation to the cultures; (**2**) LED panel; (**3**) culture bottles.

The C+, PE and SE bottles were inoculated with the previously harvested biomass pellets, giving an initial average biomass concentration in terms of dry weight (DW) of approximately 1 g_DW_ L^−1^. The cells were cultured until the stationary growth phase was reached. Therefore, the experiments had a duration of 7 d for the SE and C-SE bottles and 9 d for the C+, C-PE and PE bottles at room temperature, 21 ± 1 °C, and in continuous light (provided by an LED panel), with an average intensity of 42 µmol m^−2^ s^−1^. Agitation and CO_2_ supplementation were performed by bubbling atmospheric air, filtered through 0.22 µm cellulose acetate membranes (Orange Scientific, Braine-l’Alleud, Belgium), at a flow rate of 1.5 L min^−1^, using AP-180 air pumps from Trixie (Flensburg, Germany). The experimental setup is presented in Figure 2.

The temperature and pH of the cultures were evaluated daily, using a Consort’s C6010 electrochemical analyser (Brussels, Belgium), and light intensity was measured on the first and last days of each experiment, using a portable photo/radiometer (Delta OHM HD2102.2). To ensure that the experimental conditions were similar in each bottle, the pH of the microalgal cultures was adjusted daily using a 10% (*v*/*v*) HCl solution to match the value of the negative controls. Furthermore, the colour and turbidity of the cell-free culture media were determined on the first and last days of each experiment. Table S2 of the SM presents a summary of the samples collected from all the bottles for each assay, as well as the collection days and sample volume.

### 2.4. Microalgal Growth Monitoring in Urban Effluents

To evaluate *C. vulgaris* growth, 5 mL samples were collected once a day to measure the culture’s optical density (OD) at 680 nm, using a V-530 UV/VIS spectrophotometer from Jasco (Tokyo, Japan). To build a calibration curve between OD and biomass concentration in terms of dry weight (g_DW_ L^−1^), 25 mL samples were collected. The samples were inserted in previously dried and weighed porcelain crucibles, 10 mL for each sample (in duplicate), and dried for 24 h at 105 °C. After this period, the crucibles were allowed to cool to room temperature in a desiccator and weighed. The concentration of biomass in terms of dry weight corresponds to the mass loss in the drying process, divided by the sample volume.

The specific growth rate, µ (d^−1^), in the exponential growth phase was calculated according to Liang et al. [36], using Equation (1), where X_2_ and X_1_ represent the biomass concentration, in g_DW_ L^−1^, at times t_2_ and t_1_, in days, which correspond to the end and beginning of the exponential growth phase, respectively.

(1) $$\mu =\frac{{\ln(X}_{2}{\;/X}_{1})}{{(t}_{2}-{\;t}_{1})}$$

Biomass productivities, P_X_, were determined as described in Equation (2) for each pair of consecutive points, where X_z_ corresponds to the biomass concentration at time t_z,_ and X_z+1_ represents the biomass concentration at time t_z+1_. Maximum biomass productivities, P_X, max_ (g_DW_ L^−1^ d^−1^), correspond to the maximum values determined by this equation. The average biomass productivities, P_X, avg_ (g_DW_ L^−1^ d^−1^), were determined as indicated in Equation (3), where X_f_ and X_0_ represent the biomass concentration, in g_DW_ L^−1^, at times t_f_ and t_0_, in days, which correspond to the end and the beginning of each experiment, respectively.

(2) $$P_{X}=\frac{X_{z+1}-{\;X}_{z}}{t_{z+1}-{\;t}_{z}}$$

(3) $$P_{X,\;\mathrm{avg}}=\frac{X_{f}-{\;X}_{0}}{t_{f}-{\;t}_{0}}$$

On the last day of the experiments, the remaining culture from each bottle was centrifuged at 12,000 rpm (22,114× *g*), 20 °C, for 10 min (using a Beckman Avanti J-25 centrifuge), and the pellet/biomass was frozen at −80 °C, lyophilised, and stored in amber glass flasks until further analysis. These samples were used to determine the siloxane concentration in the biomass, as described in Section 2.6.

### 2.5. Nutrient Removal

To assess nutrient removal, nitrogen, phosphorus, COD, DOC, DIC and TDC levels were determined within the cultivation time, according to the methodologies described in Section 2.2. While in the C+, C-SE and SE assays, nitrogen concentration was determined based on NO_3_-N, for the C-PE and PE experiments, the measurements were performed directly in the organic carbon analyser, since N was in its reduced form in the assays using the PE. Phosphorus concentration over time was determined based on the PO_4_-P levels, since it was in its oxidised form in all the experimental conditions. Samples were collected in duplicate from each bottle, centrifuged at 4000 rpm (1707× *g*) for 10 min, at 20 °C, using an Eppendorf 5804 R centrifuge (Hamburg, Germany), and the supernatant was frozen at −20 °C and kept until further analysis. DOC, DIC, TDC and COD samples were collected with less frequency in assays C-SE and SE, since the organic load was much lower in these experiments compared to the assays with PE. Mass removal of nitrogen and phosphorus, MR, was calculated according to Equation (4), where $S_{0}$ corresponds to nutrients concentration at the beginning of each experiment, in mg L^−1^, and $S_{f}$ corresponds to the final nutrients concentration, in mg L^−1^. Nutrient removal efficiencies (RE) were determined as indicated in Equation (5).

(4) $${\mathrm{MR}=S}_{0}-{\;S}_{f}$$

(5) $$\mathrm{RE}\;(\%)=\frac{S_{0}-{\;S}_{f}}{S_{0}}\;\times \;100$$

The experimental data regarding nitrogen and phosphorus removal over time were fitted to the modified Gompertz model, as represented in Equation (6), where k is the nutrients uptake rate (d^−1^) and λ is the lag time (d) [37]. These kinetic parameters were calculated by minimising the sum of squared residuals using the Solver supplement of Microsoft Excel. The quality of the models was evaluated by calculating the coefficient of determination (R^2^), and the root mean squared error (RMSE). R^2^ and RMSE were calculated according to Equations (7) and (8), where the variable y corresponds to the experimental values, $\hat{y}$ to the predicted model values, $\bar{y}$ to the average experimental values and n to the data size. The closer the parameters R^2^ and RMSE are to 1 and 0, respectively, the higher the quality of the model fit.

(6) $${S\;(t)=S}_{i}+{(S}_{f}-S_{i})\;\times \;\exp\;[-\exp\;[\;k\;(\lambda -t)+1\;]]$$

(7) $$R^{2}=1-\frac{\sum_{i=1}^{n}{\left(y_{i}-\hat{y_{i}}\right)}^{2}}{\sum_{i=1}^{n}{\left(y_{i}-\bar{y}\right)}^{2}}$$

(8) $$\mathrm{RMSE}=\sqrt{\frac{\sum_{i=1}^{n}{{(y}_{i}-\hat{{\;y}_{i}})}^{2}}{n}}$$

### 2.6. Siloxanes Extraction and Quantification

#### 2.6.1. Gas Samples

Gas samples were collected from the headspace of the bottles on the first and last days of each experiment, using 1-L Tedlar air sampling bags from Supelco. A gas chromatography–ion mobility spectrometry device (GC-IMS-SILOX) from GAS (Dortmund, Germany) was used to quantify the concentrations of various individual VMSs (L3, L4, L5, D3, D4 and D5). This device provided very similar results between duplicates in the previous testing; therefore, only a single measurement was performed for each sample.

#### 2.6.2. Water Samples

From each experiment, 70 mL water samples were collected and centrifuged at 4000 rpm (1707× *g*) for 10 min, at 20 °C, using an Eppendorf 5804 R centrifuge. The supernatant (water or cell-free culture medium) was collected, frozen at −20 °C, and kept until further analysis. To extract the siloxanes from the water samples, a liquid-liquid extraction procedure was performed in duplicate. Firstly, 30 mL of each sample was transferred to a 50 mL polypropylene conical bottom centrifuge tube and subsequently spiked with 25 µL of a 5 mg L^−1^ M4Q standard in acetone (IS). Afterwards, the samples were homogenised by 1 min of vortexing and settled for 30 min. Then, 10 mL of hexane was added to the tubes, and the mixture was vortexed for 5 min, sonicated in a 720-W JP Selecta ultrasonic bath (Barcelona, Spain) for 10 min, and centrifuged for 5 min at 4000 rpm (2760× *g*) using a Hettich KG D-78532 centrifuge (Tuttlingen, Germany). The organic phase (supernatant) was then transferred to a 12 mL amber glass vial and reduced under a gentle nitrogen stream to a volume of 0.5 mL. The remaining extract was then transferred to a 1.5 mL amber glass vial and stored at −20 °C until further analysis. Laboratory blanks (LBs) were prepared following the same procedure, but without the sample addition step.

#### 2.6.3. Microalgal Biomass Samples

To evaluate the siloxane concentration in the lyophilised biomass samples, a QuEChERS extraction was performed in duplicate, adapted from the protocols developed for similar matrices (e.g., Rocha et al. [38]). Due to limitations in the lyophilised biomass obtained from each experiment, in the first step of the QuEChERS extraction procedure, approximately 1 g of biomass for the SE assay and 0.7 g for the C+ and PE assays were weighed into a 50 mL polypropylene conical bottom centrifuge tube, designated QuEChERS 1. This tube contained 2.5 g of anhydrous MgSO_4_ to remove water residues, spiked with 100 µL of a 5 mg L^−1^ solution of M4Q in hexane. After the mixture was vortexed, the first extraction step was performed by adding 5 mL of hexane, followed by 1 min of vortexing, sonication (using a 720 W JP Selecta ultrasonic bath) for 10 min, and centrifugation for 5 min at 4000 rpm (2760× *g*). The organic layer (supernatant) was collected and transferred to the second tube, designated QuEChERS 2, which contained 300 mg of MgSO_4_, 300 mg of PSA-bonded silica and 50 mg of C18. The second and third extraction steps were performed by adding 5 mL of hexane:dichloromethane (1:1) and 5 mL of hexane:ethyl acetate (1:1) to the pellet, respectively, both followed by 1 min of vortexing, sonication for 10 min, and centrifugation for 5 min at 4000 rpm (2760× *g*). After this step, the supernatant was also transferred to the QuEChERS 2 tube. The last step consisted of a dispersive solid-phase clean-up step to remove undesired compounds. The QuEChERS 2 tube containing the organic layer was vortexed for 1 min, centrifuged for 5 min at 4000 rpm (2760× *g*), and the supernatant was transferred to a 12 mL amber glass vial. The sample volume was reduced to 1 mL under a gentle nitrogen stream and stored in an amber glass vial at −20 °C until further analysis. LBs were prepared following the same procedure but without adding the microalgal biomass.

#### 2.6.4. GC Analyses for Siloxane Quantification

The siloxane quantifications in water and biomass samples were performed using a Varian Ion Trap GC–MS system (Walnut Creek, CA, USA), equipped with a 4000-GC gas chromatograph, a 240-MS ion trap mass spectrometer, a CP-1177 split/splitless injector and a CP-8410 auto-sampler. The injector was adapted with a Merlin microseal system instead of the typical silicone rubber septa to avoid VMSs contamination. Compound separation was accomplished in a low-bleed Agilent DB-5 ms ultra-inert column (30 m length × 0.25 mm diameter, 0.12 μm film thickness) at a constant flow of helium (1.0 mL min^−1^). This column avoids bleeding (natural degradation of the stationary phase) and minimises background VMS contamination. The following temperature program was used: 35 °C held for 5 min, raised at 10 °C min^−1^ until 95 °C, then 5 °C min^−1^ until 140 °C min^−1^ and, lastly, 35 °C min^−1^ until 300 °C (held for 5.43 min), in a total runtime of 30 min. The 1 μL injection volume was in split mode, with a split ratio of 100. The temperatures of the manifold, ion trap, transfer line, and injector were 50, 200, 250 and 200 °C, respectively. The mass spectrometer was operated in the electron ionisation mode (70 eV), and for quantitative analysis of target compounds, the time-scheduled selected ion storage mode was applied. The filament emission current was 10 μA. This equipment was used to analyse the following VMSs: D3, D4, D5, D6, L3, L4 and L5. The equations used to calculate siloxane concentration, as well as the calibration curves corresponding to each siloxane for both water and biomass samples, are presented in Tables S3 and S4 of the SM. It is important to note that to avoid external siloxane contamination, this work was developed in an environment in which PCPs were avoided by all members of the laboratory, detergents with siloxanes were not used, nitrile gloves wore worn at all times, all the non-graduated glass material was rinsed with distilled water and acetone, and was baked for 4 h at 400 °C before usage.

### 2.7. Statistical Analysis

Average and standard deviation (SD) values were determined for each parameter. To evaluate statistically significant differences between each experiment, Student’s paired *t*-test was used. This analysis was performed using Microsoft Excel 2021. For the kinetic parameters from the modified Gompertz models, a one-way analysis of variance (ANOVA) was used to analyse differences among the mean values. Statistical tests were performed at a significance level of 0.05.

## 3. Results and Discussion

### 3.1. Microalgal Growth in Urban Effluents

The pH and temperature variations over time can be found in Figure S1 of the SM. Over the course of the experiment, the temperature values remained constant and near 21 °C, meaning that the light supplied to the cultures did not affect the temperature in the bottles. The initial pH values were identical between the cultures grown in effluents and the respective negative controls, meaning that the addition of the inoculum did not affect the initial pH of the cultures. However, the pH values of the negative control bottles were higher than the respective effluents. This increase might be justified by the removal of organic matter during the filtration process, which altered some of the effluent’s physicochemical characteristics. Around day 2, the microalgal cultures’ pH started to increase; therefore, pH adjustments had to be performed throughout each experiment. This is a common occurrence in microalgal cultures, since, during photosynthesis, these microorganisms consume CO_2_, which accumulates mainly as HCO_3_^−^. Steady-state usage of HCO_3_^−^ as the original carbon source for photosynthesis leads to the accumulation of OH^−^ in the cells. Therefore, to neutralise these ions, there is an increase in H^+^ uptake from the culture medium, which leads to a rise in its pH [39].

**Figure 3:**
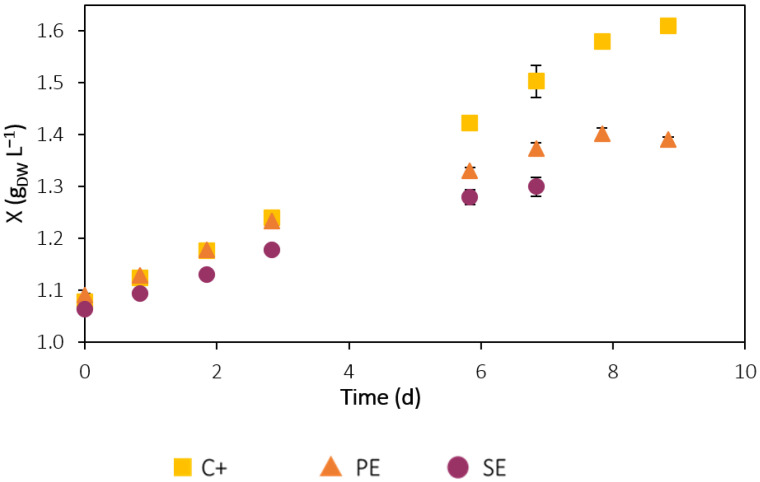
*Chlorella vulgaris* growth curves obtained for the C+, PE, and SE assays. Error bars correspond to the standard deviation of the mean obtained from two independent experiments.

Figure S2 of the SM presents the OD values over time for each experiment. The OD of the microalgal cultures gradually increased throughout the assays, but it remained close to zero for the negative control bottles, since there was no microalgal growth in these experiments. Using the data from the calibration curve that plots the OD of the microalgal cultures at 680 nm against biomass concentration in g_DW_ L^−1^, *C. vulgaris* growth curves in the C+, PE and SE were obtained, represented in Figure 3. Overall, the results show that this microalga grew successfully in all the experimental conditions. The microalgal lag phase was non-existent in all experiments, and by days 6, 7 and 8, the SE, PE, and C+ cultures, respectively, were no longer in the exponential growth phase, and the deceleration or stationary phase was reached. The differences in the duration of the exponential growth phase are related to nitrogen and phosphorus availability in each experiment: a higher concentration of nutrients leads to longer exponential growth phases.

Table 2 shows the growth parameters determined for *C. vulgaris* in each experiment. The specific growth rates were not statistically different (*p* > 0.05) between the PE and SE assays (0.034 ± 0.001 d^−1^ and 0.036 ± 0.002 d^−1^, respectively), but were significantly lower (*p* < 0.05) than C+ (0.0472 ± 0.0005 d^−1^). The same correlation was found for the average/maximum biomass productivities: 0.034 ± 0.001/0.054 ± 0.003 g_DW_ L^−1^ d^−^^1^ and 0.035 ± 0.003/0.050 ± 0.009 g_DW_ L^−^^1^ d^−^^1^ for assays with PE and SE, respectively, and 0.060 ± 0.002/0.08 ± 0.02 g_DW_ L^−^^1^ d^−^^1^ for C+. The maximum biomass concentration was the only growth parameter in which assays with PE and SE showed a significant difference. In the experiment with PE, a statistically higher (*p* < 0.05) maximum biomass concentration was reached, 1.40 ± 0.01 g_DW_ L^−1^, compared to the assay with SE, 1.30 ± 0.02 g_DW_ L^−^^1^. This difference was most likely due to the higher nutrient concentration of the PE, since higher nitrogen and phosphorus availability leads to faster and higher microalgal growth. Overall, the higher growth parameters for the positive control are explained by the optimal and adequate composition of the modified OECD culture medium for microalgal growth and higher nutrient concentration compared to the urban effluents. Furthermore, the effluents are likely to contain potentially inhibitory compounds for microalgal growth.

The colour and turbidity measurements of the cell-free culture media at the beginning and end of each experiment are presented in Table A1 of Appendix A. Wastewaters usually have high turbidity and colour due to highly diffusive particulate matter and coloured substances [40]. This causes a limitation in the light path in PBRs, decreasing photosynthetic photon flux density and reducing the photosynthetic efficiency [41]. Consequently, reduced biomass production and microalgal growth are verified. Compared to raw effluents, the lower values in the filtered effluents show that the filtration process led to a significant decrease in colour and turbidity. On the one hand, the modified OECD medium showed lower turbidity than the filtered PE, but identical to the filtered SE. Overall, the turbidity values in the filtered effluents were low, and since the growth was very similar between the SE and PE assays, this parameter does not appear to have influenced microalgal growth, contrarily to the filtered effluent’s colour, which was much higher in the effluents than the OECD medium, the latter presenting values below the limit of quantification (LOQ). Therefore, the photosynthetic efficiency was most likely higher in the C+ assay, contributing to the higher growth in this experiment.

Analysing the colour and turbidity variations from the beginning to the end of each experiment, it is possible to observe that in the C-PE assay, there was a statistically significant decrease (*p* < 0.05) in both parameters, while in the C-SE assay, turbidity and colour remained constant. This might have been due to the presence of other microorganisms in the PE, which consumed some organic matter (as discussed in Section 3.2) and possibly coloured substances, reducing colour and turbidity. In the PE assay, the colour remained constant (*p* > 0.05), and turbidity decreased significantly (*p* < 0.05), most likely as a result of the removal of organic matter by the combined action of *C. vulgaris* and the microorganisms present in the effluent. In the SE assay, the turbidity remained constant, but there was a statistically significant increase in colour (*p* < 0.05), which could be explained by the presence of microalgal cells in the samples, owed to a poor separation between the pellet and the supernatant.

Table 3 summarises a few studies on real wastewater treatment using *C. vulgaris*, focusing on the removal of nutrients, such as nitrogen, phosphorus and carbon (in the forms of DOC and COD), in comparison with the present study. The specific growth rates in this study were lower than the ones obtained by Znad et al. [5] when growing *C. vulgaris* in municipal secondary (0.62 d^−1^) and primary effluents (1.2 d^−1^) with similar nutrients concentrations. This difference could be explained by the higher inhibitory effect of the effluents used in the present study and differences in the culturing conditions, such as the lower light intensity (in the present study, the cultures were supplied with a light intensity of 42 µmol m^−2^ s^−1^, whereas in the reference study, the cultures were supplied with an intensity 180 µmol m^−2^ s^−1^), resulting in lower photosynthetic efficiency. These results were also lower than those reported by AlMomani and Ormeci [42]: 0.61 d^−1^ for a primary effluent and 0.52 d^−1^ for a secondary effluent. However, the initial biomass concentrations and cultivation times used in the present study were much lower compared to the reference study, which could explain these discrepancies. Despite these expected growth differences, the results show that *C. vulgaris* grew successfully in the urban effluents, making them a suitable culture medium for this microalga.

**Table 3:** Comparison between growth parameters and removal efficiencies of nitrogen (N), phosphorus (P), and organic matter (TDC and COD) obtained in this study and other studies focusing on real wastewater treatment using *Chlorella vulgaris*.

Type of Wastewater	Parameter	Initial Concentration	RE (%)	Growth Parameters	Cultivation Period (Days)	Reference
Highly concentrated municipal wastewater (centrate)	TN	134 mg L^−1^	>50 *	µ: 0.293 d^−1^ P_X, avg_: 0.121 g L^−1^ d^−1^	3	[7]
	PO_4_-P	212 mg L^−1^	>65 *			
	DOC	960 mg L^−1^	>82 *			
	COD	2324 mg L^−1^	>74 *			
Filtered municipal raw wastewater (influent)	TN	6.64 mg L^−1^	92	µ: 0.36 d^−1^	7	[6]
	TP	0.15 mg L^−1^	82			
Filtered primary wastewater	NH_4_-N	40.8 mg L^−1^	61	µ: 0.61 d^−1^ P_X, avg_: 0.09 g L^−1^ d^−1^	28	[42]
	TP	10.0 mg L^−1^	35			
	COD	242 mg L^−1^	40			
Filtered secondary wastewater	NO_3_-N	18.0 mg L^−1^	22	µ: 0.52 d^−1^ P_X, avg_: 0.06 g L^−1^ d^−1^		
	TP	26.0 mg L^−1^	12			
	COD	59.0 mg L^−1^	49			
Urban wastewater at 30% (*v*/*v*)	TN	59.3 mg L^−1^	88	µ: 1.06 d^−1^ P_X, avg_: 0.19 g L^−1^ d^−1^	10	[43]
	TP	9.61 mg L^−1^	98			
Filtered primary domestic wastewater at 0.02% (*v*/*v*)	TN	2.70 mg L^−1^	85	µ: 0.30 d^−1^ P_X, avg_: 0.041 g L^−1^ d^−1^	12	[44]
	TP	24.2 mg L^−1^	35			
Filtered secondary urban wastewater	NH_4_-N	0.44 mg L^−1^	100	X_f_: 1.167-1.575 g L^−1^	21	[45]
	COD	38.5 mg L^−1^	100			
Filtered municipal primary wastewater	TN	30.6 mg L^−1^	100	µ: 1.2 d^−1^ X_max_: 1.62 g L^−1^	13	[5]
	TP	6.60 mg L^−1^	80			
Filtered municipal secondary wastewater	TN	11.8 mg L^−1^	83	µ: 0.62 d^−1^ X_max_: 1.16 g L^−1^		
	TP	5.60 mg L^−1^	100			
Filtered urban primary wastewater	TN	25 mg L^−1^	86	µ: 0.034 d^−1^ X_max_: 1.40 g L^−1^ P_X, avg_: 0.034 g L^−1^ d^−1^ P_X, max_: 0.054 g L^−1^ d^−1^	9	This study
	PO_4_-P	3.0 mg L^−1^	80			
	DOC	63 mg L^−1^	79			
	COD	87 mg L^−1^	37			
Filtered urban secondary wastewater	NO_3_-N	11 mg L^−1^	52	µ: 0.036 d^−1^ X_max_: 1.30 g L^−1^ P_X, avg_: 0.035 g L^−1^ d^−1^ P_X, max_: 0.050 g L^−1^ d^−1^	7	
	PO_4_-P	2.4 mg L^−1^	87			
	DOC	10 mg L^−1^	na			
	COD	23 mg L^−1^	na			

* estimated values; COD: chemical oxygen demand; DOC: dissolved organic carbon; na: not applicable; NH_4_-N: ammonium-nitrogen; NO_3_-N: nitrate-nitrogen; PBRs: photobioreactors; PO_4_-P: phosphate-phosphorus; P_X, avg_: average biomass productivity; TN: total nitrogen; TP: total phosphorus; RE: removal efficiency; X_f_: final biomass concentration; X_max_: maximum biomass concentration; µ: specific growth rate.

### 3.2. Nutrients Removal

#### 3.2.1. Nitrogen and Phosphorus

**Figure 4:**
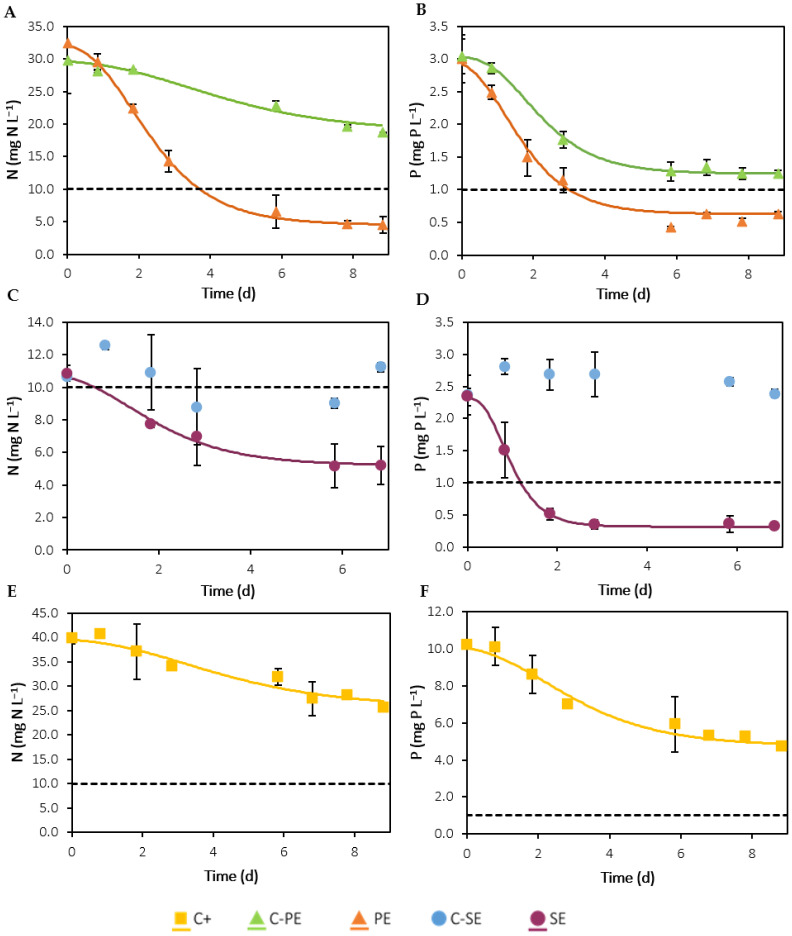
Time-course evolution of nitrogen (**A**,**C**,**E**) and phosphorus (**B**,**D**,**F**) concentration in assays with C-PE/PE (**A**,**B**), C-SE/SE (**C**,**D**) and C+ (**E**,**F**). Error bars correspond to the standard deviation of the mean obtained from two independent experiments. The filled lines represent the model fit of the modified Gompertz model to the experimental data. The horizontal dashed lines correspond to the EU legislation limits in discharged effluents: 10 mg N L^−1^ and 1 mg P L^−1^.

According to the European Union (EU) directives 1991/271/EEC and 1998/15/EC, for WWTPs with over 100,000 population equivalents, the limits for nutrient concentrations in discharged effluents are: (i) 10 mg N L^−1^ for TN, or a minimum percentage of reduction of 70–80%; (ii) 1 mg P L^−1^ for TP, or a minimum reduction percentage of 80%; and (iii) 125 mg O_2_ L^−1^ for COD, or a minimum reduction percentage of 75% [46,47]. Thus, the potential of *C. vulgaris* to remove nitrogen and phosphorus from the urban effluents and reduce the nutrients levels to values below these legislation limits was evaluated. Figure 4 presents the time-course evolution of nitrogen and phosphorus concentration in each experiment and the fitting of the modified Gompertz model to the experimental data. Table 4 presents the initial nutrients concentrations, removal parameters, and kinetic parameters determined through this model. The results show that when cultured in urban effluents, *C. vulgaris* can efficiently remove nitrogen and phosphorus, reaching values below the EU legislation limits within the cultivation time. Overall, the modified Gompertz model appears to reflect a good adjustment to the experimental data, since RMSE values were equal to or below 1.265 mg L^−1^ and R^2^ values were equal to or above 0.945. In both effluents, the molar N:P ratios were within the optimal range (5 to 30) for microalgal growth [48].

The initial nitrogen concentration was statistically higher (*p* < 0.05) in the modified OECD medium than in the filtered effluents. However, the difference was mild compared to the PE, but nearly four times higher than in the SE. In the C-PE assay, a nitrogen mass removal of 11.06 ± 0.02 mg L^−1^ was observed, which might have been due to the action of microorganisms present in the effluent. However, after 9 days, the nitrogen concentration was still above the legislation limit (Figure 4A). A significantly higher (*p* < 0.05) mass removal was observed in the PE assay compared to the remaining experiments: 28 ± 1 mg L^−1^. Comparing these results with the C-PE experiment, there was a significant improvement in nitrogen removal when *C. vulgaris* was present, since the mass removal nearly tripled in these conditions. Moreover, after 6 days, the concentration levels were below the legislation limit, as shown in Figure 4A. Comparing the mass removal in the C-PE assay with the C+ assay (14.1 ± 0.3 mg L^−1^), the sum of these results is in accordance with the mass removal in the PE assay. This correlation indicates that the improvement in the PE compared to the C-PE resulted from the combined action between *C. vulgaris* and the microorganisms in the effluent. In the C-SE assay (Figure 4C), nitrogen concentration remained approximately constant over the course of the experiment and, therefore, no removal was observed. The lowest mass removal (5.66 ± 0.01 mg L^−1^) and lowest culture time necessary to reach the legislation limit (2 d) were observed for the SE assay (Figure 4C), which can be explained by the lower initial nitrogen concentration present in this assay. In terms of kinetic parameters for nitrogen removal, the mean uptake rates and lag times were not statistically different (*p* > 0.05) between all the experiments, according to the ANOVA statistical test.

The initial phosphorus concentrations were statistically higher (*p* < 0.05) in the modified OECD medium than in the filtered effluents, and, therefore, the mass removal was also higher in these experimental conditions. In the C-PE experiment (Figure 4B), besides nitrogen removal, phosphorus concentration also decreased 1.8 ± 0.2 mg L^−1^. A possible explanation for this removal is the presence of polyphosphate accumulating organisms (PAO) in the PE effluent, bacteria that accumulate phosphorus in the form of polyphosphates within the cells [49]. However, an even higher removal was observed in the PE assay, 2.5 ± 0.1 mg L^−1^, showing once again that the introduction of microalgae greatly improves the removal of nutrients. Moreover, as opposed to the respective negative control, concentrations below the legislation limit were achieved within the cultivation time for the PE assay, after 6 days (Figure 4B). Phosphorus concentration remained constant throughout the C-SE assay (Figure 4D), but a mass removal of 2.02 ± 0.02 mg L^−1^ was observed in the SE experiment due to bioassimilation by microalgae. There was no statistical difference between the mass removal and the removal efficiencies in the SE and PE assays, since the initial phosphorus concentrations were identical. However, the SE cultures achieved concentrations below the legislation limit after only 2 days (Figure 4D), sooner than the PE cultures (statistically different uptake rates; *p* < 0.05). The uptake rate in the SE assay was higher, which can be related to the lower nutrient concentrations in this effluent. Initially, this experiment had a lower nitrogen and phosphorus availability for the same biomass concentration and cell number. This nutrient limitation might have induced microalgae to quickly assimilate phosphorus in the SE assay, resulting in a higher uptake rate. No statistical difference was found between the average lag times of each experiment.

Nitrogen removal efficiencies for PE (86%) and SE (52%) were lower than those obtained by Znad et al. [5] for the filtered municipal primary (100%) and secondary (83%) effluents, with initial nitrogen concentrations of 30.6 and 11.8 mg L^−1^, respectively. In the mentioned study, a mass removal of 30.6 mg L^−1^ was obtained for the primary effluent, which is not very different from the mass removal obtained in the PE assay from the present work, 27.9 mg L^−1^. However, the mass removal was lower in the SE assay, 5.66 mg L^−1^, compared to the value determined by Znad et al. [5], 9.79 mg L^−1^. A possible justification for these results could be the lower light intensity used in the present study, resulting in lower photosynthetic efficiency and, consequently, lower growth and nutrient consumption. Regarding phosphorus removal, Znad et al. [5] obtained removal efficiencies of 80% for the primary effluent, identical to the removal in the PE assay, and 100% for the secondary effluent, higher than the SE experiment (87%). In terms of mass removal, the values were much higher in the mentioned study. These differences might be due to the higher initial phosphorus concentrations of 6.6 and 5.6 mg L^−1^ for primary and secondary effluents, respectively, compared to this study: 2.5 mg L^−1^ in the PE and 2.0 mg L^−1^ in the SE.

#### 3.2.2. Carbon

**Figure 5:**
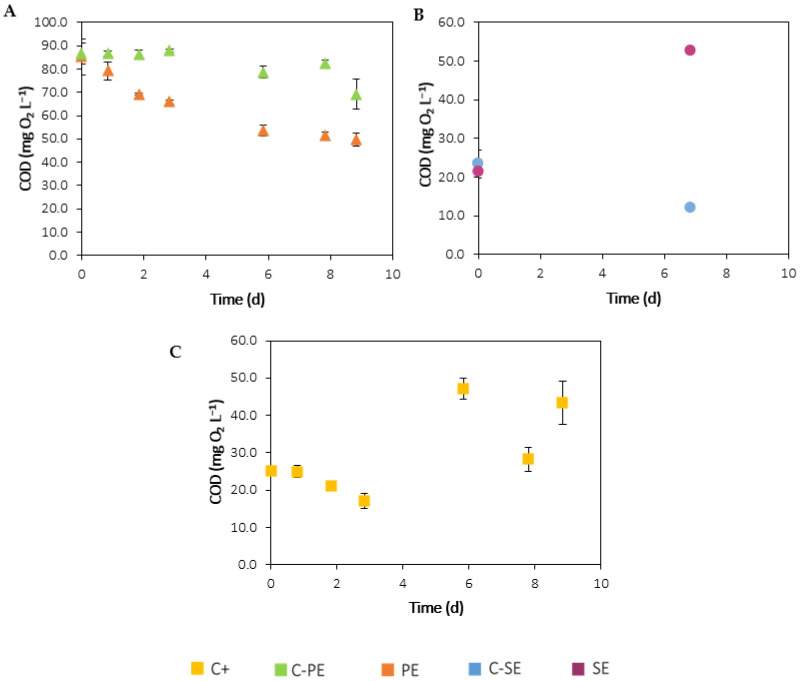
Time-course evolution of COD in assays with C-PE/PE (**A**), C-SE/SE (**B**), and C+ (**C**). Error bars correspond to the standard deviation of the mean obtained from two independent experiments.

**Figure 6:**
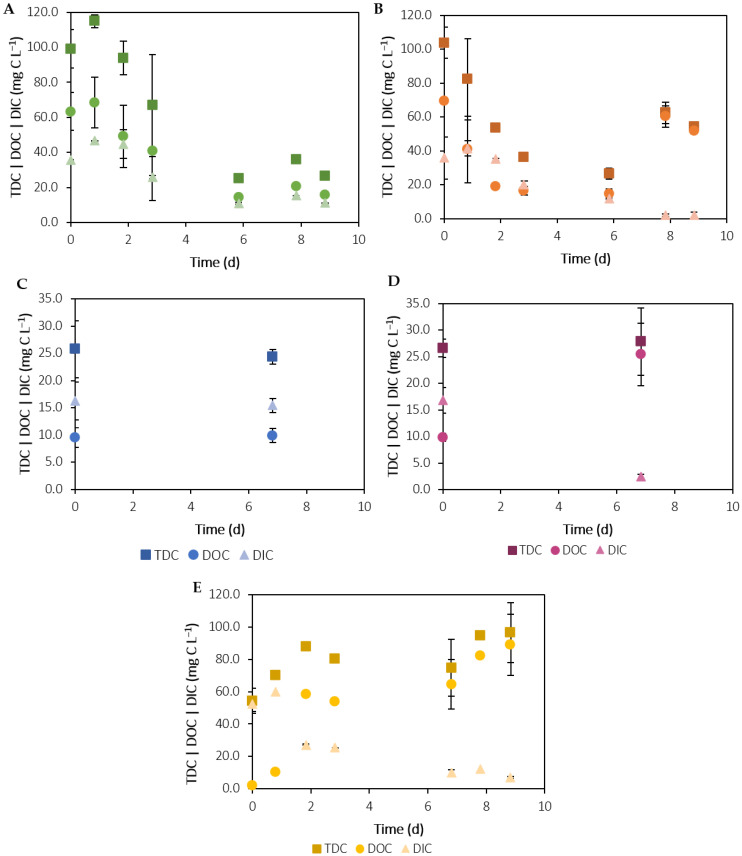
Time-course evolution of TDC, DOC and DIC in assays with C-PE (**A**), PE (**B**), C-SE (**C**), SE (**D**) and C+ (**E**). Error bars correspond to the standard deviation of the mean obtained from two independent experiments.

Due to its mixotrophic metabolism, *C. vulgaris* can perform mixotrophy and utilise different forms of carbon: inorganic carbon for photosynthesis and organic carbon for respiration [50]. Figure 5 and Figure 6 present the time-course evolution of COD and DIC/DOC/TDC, respectively, in each experiment. In the C+ assay, the initial DOC concentration was extremely low, 2 ± 1 mg L^−1^, as opposed to the DIC, 52 ± 4 mg L^−1^ (Figure 6E). These results are in accordance with the inorganic typology of the culture medium, which contains only inorganic carbon in the form of soluble carbonates from NaHCO_3_. Over the course of this experiment, an 86% decrease in DIC was verified. This removal is explained by the consumption of inorganic carbon by microalgae during photosynthesis, through carbon-concentrating mechanisms that involve the dehydration of HCO_3_^−^ to CO_2_ [51]. In contrast, the DOC concentration increased over time, reaching concentrations as high as 89 ± 19 mg L^−1^, most likely due to the excretion of metabolic products, such as soluble microbial products and extracellular polymeric substances (EPS) [52]. These results are in accordance with the determined COD values, which represent the amount of oxygen equivalents consumed in the chemical oxidation of organic matter by potassium dichromate. As shown in Figure 5C, COD was low at the beginning of the experiment, since there was nearly no organic matter, but increased in the last days of the experiment, due to the release of the mentioned metabolites.

In both effluents, the COD values were already below the EU legislation limit (125 mg O_2_ L^−1^). Compared to the raw effluents, the lower initial DOC concentrations in the filtered effluents show that the filtration process significantly removed organic matter. This pre-treatment step is very important for microalgal cultures, since large organic solids cannot be assimilated directly by microalgae and can even hinder photosynthesis by increasing turbidity [53]. The addition of inoculum does not appear to have affected these parameters, as the initial DOC and DIC concentrations were not statistically different (*p* > 0.05) between the effluent-grown cultures and the respective negative controls. In parallel with nitrogen and phosphorus removal, DOC and DIC concentrations in the C-PE assay (Figure 6A) decreased by approximately 78% and 70% until day 6, to values within the same concentration range as in the C-SE assay, 14 ± 1 mg L^−1^ and 10.9 ± 0.5 mg L^−1^, respectively, probably due to the action of microorganisms in the effluent. In the PE assay (Figure 6B), DIC decreased approximately 94% until the end of the experiment, while DOC decreased 79% until day 6. The removal of organic matter was confirmed by a 20% decrease in COD for assay C-PE and 37% for assay PE, as shown in Figure 5A. Compared to the negative control, the slightly higher DOC and COD removal in the PE assay suggests that *C. vulgaris* grew mixotrophically in these conditions. Therefore, the decrease in organic carbon was most likely a result of the combined action of microalgae and other microorganisms possibly present in the PE. However, after day 6, DOC started increasing due to the excretion of secondary metabolites during the stationary phase of microalgal growth. No significant differences (*p* > 0.05) were verified between the DOC/DIC values in the C-SE assay (Figure 6C) on the first (10 ± 2 mg L^−1^/16 ± 3 mg L^−1^) and last (10 ±1 mg L^−1^/15 ± 1 mg L^−1^) days of the experiment. Even though there was a slight decrease in COD (Figure 5B), the values were in a low concentration range and, therefore, these results do not appear indicative of organic matter removal. In the SE assay (Figure 6D), DIC decreased from 17 ± 2 mg L^−1^ to 2.4 ± 0.5 mg L^−1^ (approximately 86%) due to the consumption of inorganic carbon for photosynthesis. On the other hand, DOC concentration increased from 9.8 ± 0.8 mg L^−1^ to 25 ± 6 mg L^−1^, as well as COD (Figure 5B), most likely due to the excretion of metabolic products.

### 3.3. Siloxanes Removal

#### 3.3.1. Gas and Water Samples

**Figure 7:**
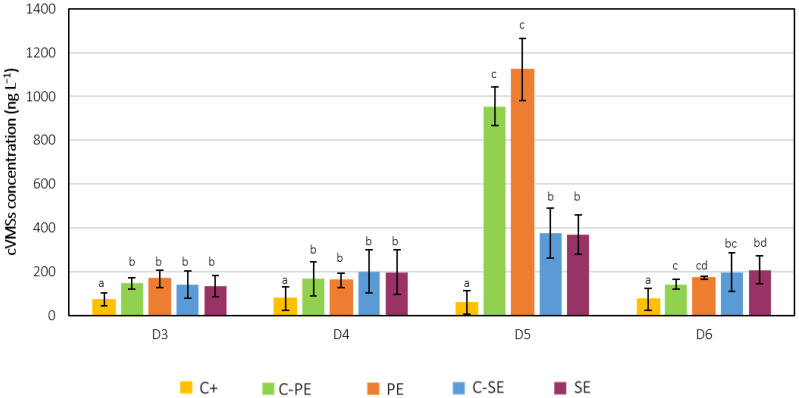
Initial D3, D4, D5 and D6 siloxane concentration in the cell-free culture medium in each experiment. Error bars correspond to the standard deviation of the mean obtained from two independent experiments. For each siloxane, average values sharing at least one common letter (a, b, c, and d) are not statistically different (*p* > 0.05).

VMSs were not detected in the collected gas samples at the beginning and end of the C-SE and SE assays. D6 is not analysed in the GC-IMS-SILOX equipment, so it was not possible to assess its presence in these samples. However, D4 was detected in concentrations between 0.06 and 0.08 mg m^−3^ on the first day, not only for the C-PE and PE experiments, but also in the C+ assay. This could be evidence of external contamination of the cultures with D4. In the cell-free culture medium or water samples, all the studied cVMSs were detected, but linear siloxanes L3 and L4 were not. Even though L5 was detected in both raw effluents at very low concentrations, no significant results were obtained for the variation of this VMS in any of the experiments and, therefore, these results were not presented. Figure 7 shows the initial cVMS concentrations in the cell-free culture medium in each experiment. Even though cautionary protocols were followed to avoid siloxane contamination, D3, D4, D5 and D6 were detected in the C+ assay and remained constant over the course of the experiment. These results can be derived from the presence of these compounds in the cultivation bottles and aeration tubes, or even from external contaminations during sample collection and pH adjustments. Nevertheless, the detected cVMS concentrations in this assay were statistically lower (*p* < 0.05) when compared to the results obtained for the effluent-grown cultures. Overall, no statistical difference was found in the cVMS concentration between the effluent-grown cultures and the respective negative controls, which suggests that the inoculum did not contain these compounds.

**Figure 8:**
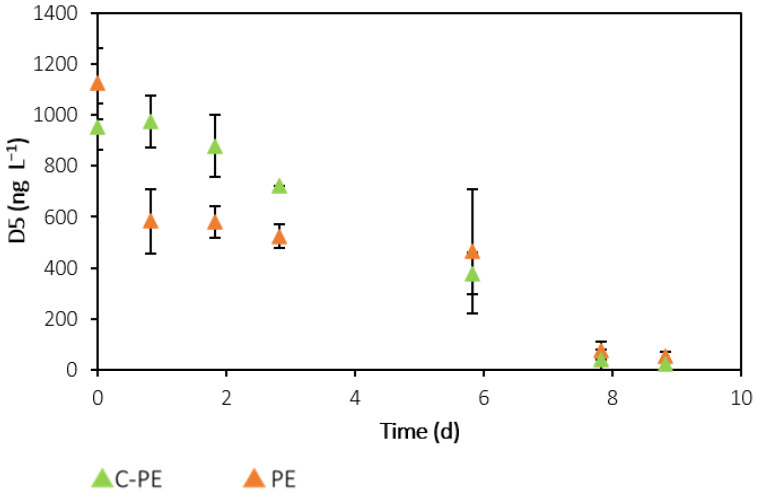
Time-course evolution of D5 concentration in the cell-free culture medium samples collected from C-PE and PE assays. Error bars correspond to the standard deviation of the mean obtained from two independent experiments.

D5 was the predominant siloxane in both PE and SE. This compound is one of the most problematic VMSs; its widespread daily use in PCPs leads to its substantial occurrence in wastewaters [21,22]. The filtration process does not appear to have altered the concentration of this compound, since no statistical difference (*p* > 0.05) was found between the raw and filtered effluents. Moreover, D5 concentrations were statistically higher in the raw PE (904 ± 83 ng L^−1^) compared to the raw SE (455 ± 141 ng L^−1^). The lower concentration in the secondary-treated effluent can be explained by the significant aeration process occurring during secondary treatment in WWTPs, which can lead to the volatilisation of over 50% of the VMSs [24]. In both C-SE and SE assays, no removal was verified, since D5 concentrations remained approximately constant over the course of the experiment, at 376 ± 115 ng L^−1^ and 368 ± 90 ng L^−1^, respectively. However, in the C-PE and PE assays, a significant decrease was observed. Figure 8 shows the evolution of D5 with time in these assays, in which an identical removal of approximately 98% was seen for both experiments. As mentioned in Section 2.3, a negative control bottle without light exposure was tested. Comparing the results obtained for these two negative controls, identical values for all VMSs were found, ruling out removal by photodegradation. Therefore, the main removal pathway for D5 was most likely volatilisation. Another hypothesis for D5 removal in the negative control could be a combination of volatilisation with D5 degradation by bacteria present in the effluent, even if the latter was at a lower extent. However, after one day of culturing, the D5 concentration in the PE assay was significantly lower than in the negative control, indicating that the presence of microalgae potentiated this removal during the first days of the experiment. D5 is a lipophilic compound with high *K_ow_* and *K_oc_* and low water solubility [23], favouring its partition from the effluents to both gas and microalgal biomass matrices. Even though aeration might be responsible for removing a high percentage of the D5 load after 9 days of culture, the presence of microalgae might be useful in cases where it is necessary to reduce D5 levels in a shorter period.

**Figure A1:**
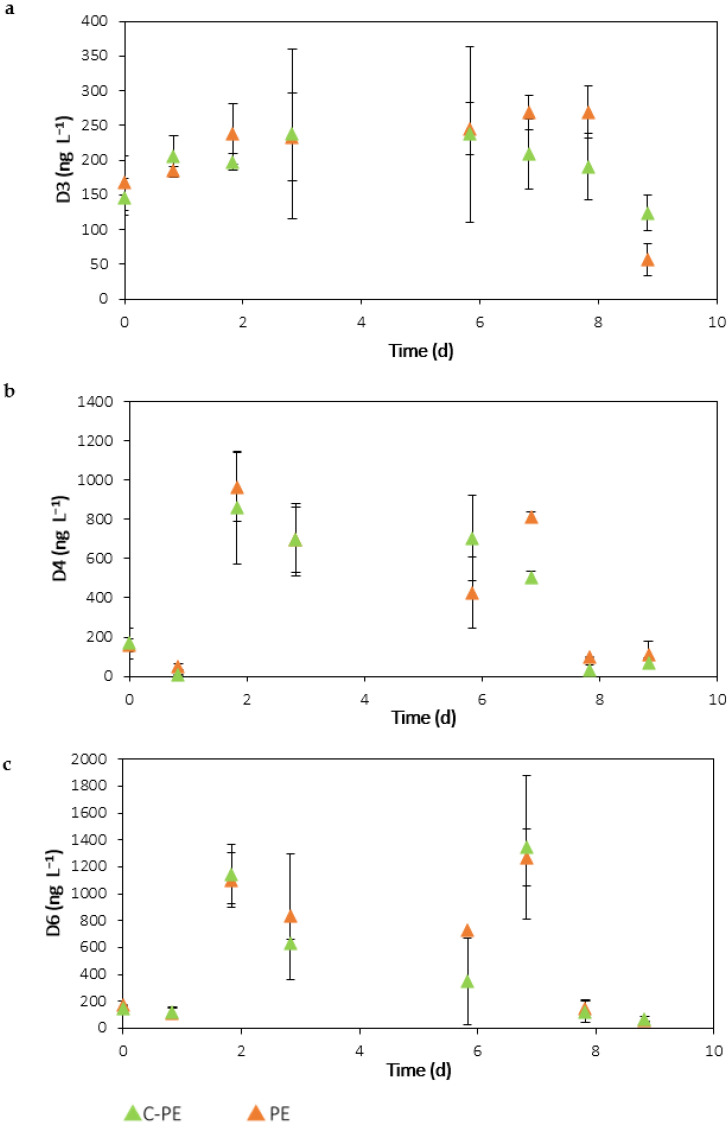
Time-course evolution of D3 (**a**), D4 (**b**) and D6 (**c**) concentrations for the C-PE and PE experiments.

D3 concentration was not statistically different (*p* > 0.05) between the raw PE (184 ± 88 ng D3 L^−1^) and SE (127 ± 35 ng D3 L^−1^), and the filtration process does not seem to have influenced the concentration of this cVMS in the effluents, as the values were statistically similar (*p* > 0.05) between the raw and filtered matrices. Time-course evolution of D3 concentration in the cell-free culture medium from the C-PE and PE assays is presented in Figure A1a of Appendix B. It is possible to observe that the average D3 concentrations did not vary significantly between each day, as they remained between 124 and 238 ng D3 L^−1^. However, due to the standard deviations associated with these mean concentrations, it is difficult to conclude on a tendency for the variation of D3 concentration over time. This variability might be related to the fact that D3 has the highest vapour pressure and lowest boiling point of the studied VMSs and, therefore, it is a highly volatile siloxane. Over the course of the C-SE and SE experiments, no significant variations were observed, and average D3 concentrations were 142 ± 63 ng D3 L^−1^ and 134 ± 49 ng D3 L^−1^, respectively.

No statistical difference was found (*p* > 0.05) between D4 and D6 concentrations in the raw PE (198 ± 85 ng D4 L^−1^ and 247 ± 21 ng D6 L^−1^) and SE (246 ± 16 ng D4 L^−1^ and 233 ± 39 ng D6 L^−1^). The filtration process does not appear to have affected the concentration of D4 in both effluents and D6 in the SE, since there was no statistical difference between the detected values in the raw and filtered effluents. However, the initial concentrations of D6 in the C-PE and PE assays were statistically lower (*p* < 0.05) than the raw effluent. D6 is the cVMS with the lowest water solubility, the highest molecular weight and the highest *K_ow_* and *K_oc_* values, and therefore this highly lipophilic compound was most likely attached to the organic matter in the PE. During the filtration process of this effluent, there was a significant removal of organic matter and, therefore, D6 might have been retained in the residue, which led to a reduced concentration in the filtered effluent. The time-course evolution of D4 and D6 concentration in the cell-free culture medium from the C-PE and PE assays is presented in Figure A1b,c of Appendix B, respectively. Even though the concentrations in the first and last two days of the experiments were similar, the concentrations for days 2, 3, 6 and 7 varied greatly throughout the experiments and between duplicate samples, without an apparent pattern. Even with all precautions, possible external contaminations with D4 and D6 could have occurred during sample collection in these days, which could justify some of the higher values. Similarly to D3 and D5, no significant variations were found in D4/D6 concentrations over the course of the C-SE and SE experiments, with average values of 201 ± 99 ng D4 L^−1^/197 ± 88 ng D6 L^−1^ and 197 ± 102 ng D4 L^−1^/208 ± 64 ng D6 L^−1^, respectively. These results might indicate that microalgae were not able to remove these siloxanes from the SE. Since variabilities were not detected in the negative control, this could suggest that volatilisation was absent. However, this seems unlikely, especially because the air was bubbled continuously through the culture, which favours the partition from the liquid to the gaseous phase. Under these circumstances, it seems that the extraction and analysis methodologies used in this study were not sensible enough to detect variations in this low range of cVMSs concentrations.

#### 3.3.2. Biomass Samples

**Figure 9:**
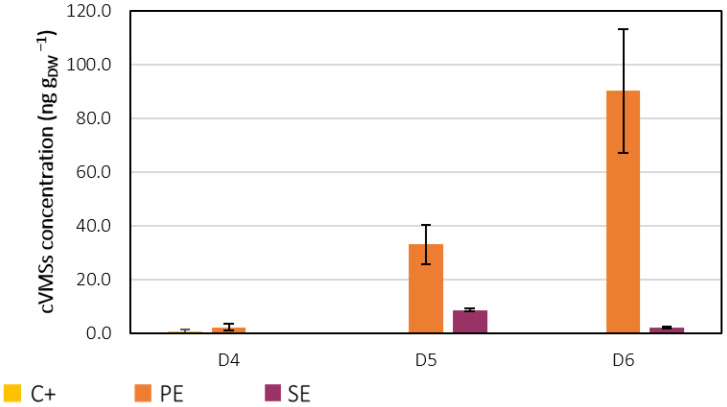
D4, D5 and D6 concentration in the lyophilised biomass samples collected from cultures C+, PE and SE.

D4, D5 and D6 were detected in the lyophilised biomass samples. During the QuEChERS extraction procedure, the sonication steps most likely contributed to cell rupture as well as the multiple centrifugation and homogenisation steps. Therefore, the detected siloxanes in the biomass samples may have been removed from the wastewaters by bioadsorption and/or bioaccumulation mechanisms. Figure 9 shows the determined cVMSs concentration in terms of the mass of siloxanes per mass of lyophilised samples. In the biomass from the C+ assay, only D4 was detected at a concentration of 0.8 ± 0.8 ng D4 g_DW_^−1^, much lower than the PE assay. The high standard deviation reflects the high variability between the two independent experiments, showing that D4 was present in these samples, most likely due to external contamination, which is in accordance with the results from the gas samples.

**Figure 10:**
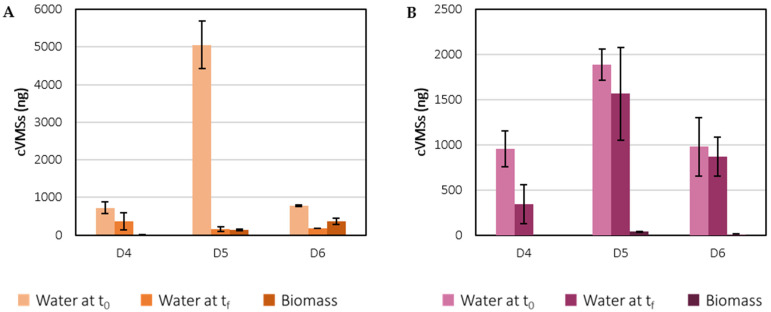
Estimated mass of siloxanes in biomass and water at the first (t_0_) and last (t_f_) days of the PE (**A**) and SE (**B**) assays.

**Figure 11:**
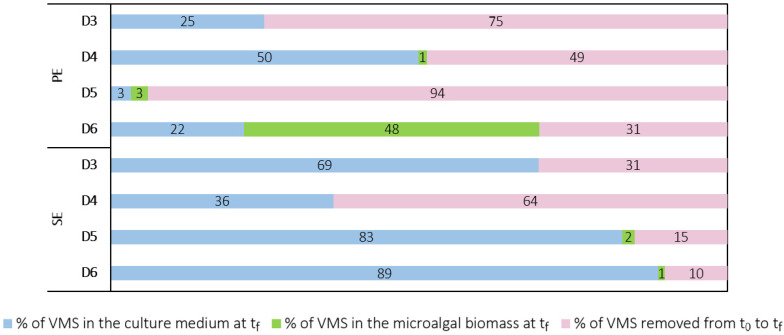
Mass distribution (%) of D3, D4, D5 and D6 between the first (t_0_) and last (t_f_) days of the PE and SE assays.

In the biomass samples from the PE assay, D4, D5, and D6 concentrations were 2 ± 1 ng D4 g_DW_^−1^, 33 ± 7 ng D5 g_DW_^−1^ and 90 ± 23 ng D6 g_DW_^−1^, respectively. Only D5 and D6 were detected in the SE assay at concentrations of 8.8 ± 0.6 ng D5 g_DW_^−1^ and 2.3 ± 0.6 ng D6 g_DW_^−1^, respectively. The amount of D5 and D6 was significantly higher in the biomass samples from the PE compared to the SE. However, to understand to what extent cVMSs accumulated in the microalgal biomass, it is necessary to compare the mass of siloxanes in the biomass from the whole culture with the initial and final amounts of siloxanes in each experiment. Figure 10 shows the estimated mass of cVMSs in the biomass as well as in the cell-free culture media (water) at the first and last days of the PE and SE experiments. These values were obtained as described in the SM. It is important to note that the discrepancies between the initial and final mass of siloxanes in the aqueous phase are not necessarily related to a decrease in their concentrations, but to a decrease in volume and mass loss due to sample collection throughout the experiments. Figure 11 presents the relative mass distribution of D3, D4, D5 and D6 in these assays.

In the PE assay, 1% of the initial amount of D4 in the filtered effluent was in the biomass, while 50% remained in the aqueous phase on the last day of the experiment. On the other hand, D4 was not found in the biomass samples from the SE assay, and 36% remained in the cell-free culture medium. For D5, on the last day of the PE experiment, approximately 3% of the initial amount of D5 was in the microalgal biomass, but only 3% remained in the aqueous phase. These results confirm that microalgae contributed to D5 removal but to a small extent, according to the time-course variation of D5 concentration in water samples from C-PE and PE assays. For the SE, an identical percentage was obtained for D5 in biomass (2%), while 83% remained in the culture medium. Lastly, in the PE assay, D6 was the VMS that accumulated to the greatest extent in the microalgal biomass, since 48% of the initial mass of D6 was detected in the biomass samples, while 22% remained in the aqueous phase. As mentioned before, D6 is the cVMS with the lowest water solubility, highest molecular weight, and highest *K_ow_* and *K_oc_* values, and therefore, it is highly lipophilic. All these physicochemical properties contribute to the higher adsorption of D6 onto the surface of microorganisms such as microalgae, compared to the remaining cVMSs in this study. In contrast, in the SE experiment, 89% of the amount of D6 in the filtered effluent was in the culture medium, while only 1% was in the microalgal biomass. Overall, these results suggest that when *C. vulgaris* was cultured in the PE, there was an absorption or accumulation of siloxanes in biomass to a greater extent, compared to the culture of the same species in the same experimental conditions, but in the SE. Therefore, the composition of the PE seems to be more suitable for the study of siloxane removal by microalgae.

This being the first time that microalgae were tested (and reported) as an alternative for removing siloxanes from wastewaters, it is difficult to precisely establish the mechanisms of VMS removal by microalgae. VMSs are lipophilic compounds with a range of volatilities from volatile to semi-volatile, and although they are not entirely hydrophobic, they tend to partition away from the aqueous media into the air or into the sludge (in the case of a WWTP facility). This suggests that the behaviour of VMS in water (or wastewater in this case) can change depending on the type of wastewater. PE is a more complex matrix than SE and typically shows more organic (and VMS) content. On the one hand, VMSs have more organic matter to compete with microalgae for the partition from water, but on the other hand, there are more VMSs available for removal, and all this organic matter is also in contact with the microalgae, facilitating the potential capture, instead of the release to the atmosphere. With SE, this release into the atmosphere can precisely be favoured by the higher water content and lower organic matter, which may explain the difference for D6 in both. Compared to D5, D6 is the cVMS with the lowest water solubility, highest molecular weight, and highest K_ow_ and K_oc_ values, and, therefore, is the most lipophilic of both. The microalgal cell surface could have a higher affinity for D6 due to all these properties, compared to the remaining VMSs, and primarily adsorb this compound while D5 is volatilised. There might not be enough microalgal cells to adsorb primarily D6 and then D5 during the culture period. As can be seen, there are still many gaps to be filled in the knowledge of VMS capture by microalgae, but this study is the first indication that this is a valid approach.

## 4. Conclusions

*C. vulgaris* grew successfully in both primary and secondary urban WWTP effluents. When cultured in these effluents, they efficiently removed nitrogen and phosphorus, reaching values below the EU legislation limits within the cultivation time. Approximately 86% of nitrogen and 80% of phosphorus were efficiently removed from the PE, most likely due to the combined action of *C. vulgaris* with microorganisms present in the effluent. In the SE culture, 52% of nitrogen and 87% of phosphorus were removed due to microalgal assimilation. In terms of VMSs removal, D5 was the predominant VMS in both PE (904 ± 83 ng D5 L^−1^) and SE (455 ± 141 ng D5 L^−1^). A decrease in concentration of approximately 98% was found in the culture medium throughout the PE assay, most likely due to volatilisation. Three out of the seven analysed VMSs were detected in the biomass samples from this assay (D4, D5 and D6), but D6 was the one that accumulated to a greater extent, since 48% of the initial mass of D6 was detected in the biomass samples. Moreover, the results suggest that when *C. vulgaris* was cultured in the PE, absorption or accumulation of siloxanes in biomass might have occurred to a greater extent, compared to the culture of the same species in the same experimental conditions, but in the SE. Therefore, the composition of the PE appears to be more suitable for the study of siloxane removal by microalgae. In conclusion, the results confirm the remarkable potential of *C. vulgaris* in the bioremediation of wastewaters, indicating a possible removal of specific VMSs from effluents, at least for those with a more lipophilic character. However, different experimental conditions must be tested to better understand this subject. For instance, culturing *C. vulgaris* at a larger scale would allow: (i) siloxane analysis in the culture medium in triplicates and a higher volume in each sample to amplify the signal and decrease variability; and (ii) higher quantity of biomass recovered at the end of the experiment, which could be useful to perform recovery tests based on the QUECHERS method. Additionally, in future experiments, optimised methods for VMS extraction from microalgal biomass can be developed and validated, since the QuEChERS extraction procedure used in the present work was an adaptation of a method developed for other (similar) matrices. If the removal of siloxanes by microalgae is possible in these conditions, the type of mechanisms involved in this process could be evaluated.

## Figures and Tables

**Table 1:** Physicochemical characterisation of the raw and filtered effluents used in this study.

Parameters	Value				Unit
	Raw PE	Filtered PE	Raw SE	Filtered SE	
pH	7.3	8.1	7.1	7.7	-
Conductivity	4 250	nd	1 822	nd	µS m^−1^
Colour	260 ± 7	76 ± 8	43 ± 1	29 ± 7	HU
Turbidity	55 ± 3	5.5 ± 0.4	2.4 ± 0.2	0.8 ± 0.2	NTU
Chemical oxygen demand (COD)	76 ± 2	87 ± 4	24 ± 2	23 ± 4	mg O_2_ L^−1^
Dissolved organic carbon (DOC)	255 *	63 ± 11	31 ± 26	10 ± 2	mg C L^−1^
Total dissolved carbon (TDC)	298 *	99 ± 11	51 ± 26	26 ± 5	mg C L^−1^
Dissolved inorganic carbon (DIC)	42 *	35.6 ± 0.2	19.66 ± 0.01	16 ± 3	mg C L^−1^
Total nitrogen (TN)	25.89 ± 0.02	25 ± 6	15 ± 1	14 ± 4	mg N L^−1^
Nitrate-nitrogen (NO_3_-N)	<LOD	<LOD	12.25 ± 0.08	10.67 ± 0.02	mg N L^−1^
Total phosphorus (TP)	2.21 ± 0.05	nd	1.63 ± 0.02	nd	mg P L^−1^
Phosphate-phosphorus (PO_4_-P)	3.0 ± 0.2	3.0 ± 0.3	2.83 ± 0.01	2.4 ± 0.3	mg P L^−1^
N:P molar ratio	26	18	10	13	-
Total solids (TS)	1.47 ± 0.02	nd	1.118 ± 0.004	nd	g L^−1^
Total volatile solids (TVS)	0.22 ± 0.01	nd	0.143 ± 0.004	nd	g L^−1^
Hexamethylcyclotrisiloxane (D3)	184 ± 88	147 ± 26	127 ± 35	142 ± 63	ng D3 L^−1^
Octamethylcyclotetrasiloxane (D4)	198 ± 85	168 ± 78	246 ± 16	201 ± 99	ng D4 L^−1^
Decamethylcyclopentasiloxane (D5)	904 ± 83	954 ± 92	455 ± 141	376 ± 115	ng D5 L^−1^
Dodecamethylcyclohexasiloxane (D6)	247 ± 21	142 ± 22	233 ± 39	197 ± 88	ng D6 L^−1^
Octamethyltrisiloxane (L3)	<LOD	<LOD	<LOD	<LOD	ng L3 L^−1^
Decamethyltetrasiloxane (L4)	<LOD	<LOD	<LOD	<LOD	ng L4 L^−1^
Dodecamethylpentasiloxane (L5)	16 ± 22	<LOD	1.7 ± 0.7	<LOD	ng L5 L^−1^

HU: Hazen units; LOD: limit of detection; nd: not determined; NTU: nephelometric turbidity unit; N:P ratio: nitrogen to phosphorus ratio; PE: primary effluent; SE: secondary effluent; *—results of one measurement.

**Table 2:** Growth parameters determined for *Chlorella vulgaris* in each experiment.

Experiment	µ (d^−1^)	X_max_ (g_DW_ L^−1^)	P_X, max_ (g_DW_ L^−1^ d^−1^)	P_X, avg_ (g_DW_ L^−1^ d^−1^)
C+	0.0472 ± 0.0005 ^a^	1.61 ± 0.01 ^a^	0.080 ± 0.002 ^a^	0.060 ± 0.002 ^a^
PE	0.034 ± 0.001 ^b^	1.40 ± 0.01 ^c^	0.054 ± 0.003 ^b^	0.034 ± 0.001 ^b^
SE	0.036 ± 0.002 ^b^	1.30 ± 0.02 ^b^	0.050 ± 0.009 ^b^	0.035 ± 0.003 ^b^

P_X, avg_: average biomass productivity; P_X, max_: maximum biomass productivity; X_max_: maximum biomass concentration; µ: specific growth rate. Values are presented as the mean ± standard deviation obtained from two independent experiments. Within the same column, average values sharing one common letter (^a^, ^b^ and ^c^) are not statistically different (*p* > 0.05).

**Table 4:** Nitrogen and phosphorus initial concentration, removal parameters, and kinetic parameters correspondent to the modified Gompertz model obtained for each experiment.

Nutrients	Experiment	S_0_ (mg L^−1^)	MR (mg L^−1^)	RE (%)	k (d^−1^)	λ (d)	R^2^	RMSE (mg L^−1^)
N	C+	40 ± 1 ^a^	14.1 ± 0.3 ^a^	35.5 ± 0.7 ^a^	0.4 ± 0.3	1 ± 2	0.945	1.265
	C-PE	29.78 ± 0.01 ^c^	11.06 ± 0.02 ^c^	37.13 ± 0.02 ^a^	0.4 ± 0.3	1 ± 2	0.971	0.752
	PE	32.4 ± 0.5 ^c^	28 ± 1 ^d^	86 ± 4 ^c^	0.8 ± 0.1	0.6 ± 0.2	0.999	0.410
	C-SE	10.67 ± 0.02 ^b^	na	na	Na	na	na	na
	SE	10.9 ± 0.5 ^b^	5.66 ± 0.01 ^b^	52.1 ± 0.1 ^b^	0.8 ± 0.7	0.2 ± 1.9	0.986	0.227
P	C+	10.2 ± 0.1 ^a^	5.5 ± 0.2 ^a^	53 ± 2 ^a^	0.6 ± 0.3	0.5 ± 1	0.972	0.347
	C-PE	3.0 ± 0.3 ^b^	1.8 ± 0.2 ^b^	59 ± 3 ^c^	0.9 ± 0.1	0.7 ± 0.3	0.997	0.039
	PE	3.0 ± 0.4 ^b^	2.5 ± 0.1 ^c^	80.0 ± 0.1 ^b^	1.0 ± 0.2	0.2 ± 0.5	0.984	0.115
	C-SE	2.4 ± 0.3 ^b^	na	na	na	na	na	na
	SE	2.3 ± 0.1 ^b^	2.02 ± 0.02 ^bc^	86.5 ± 0.9 ^b^	2.2 ± 0.2	0.3 ± 0.1	0.998	0.039

k: uptake rate; MR: mass removal; na: not applicable; RE: removal efficiency; R^2^: coefficient of determination; RMSE: root mean squared error; S_0_: initial nutrients concentration; λ: lag time. Values are presented as the mean ± standard deviation obtained from two independent experiments. Within the same column and for the same element, average values sharing at least one common letter (^a^, ^b^, ^c^ and ^d^) are not statistically different (*p* > 0.05).

## Data Availability

The data presented in this study are available in the article and in the Supplementary Material.

## Supplementary Materials

The following are available online at https://www.mdpi.com/article/10.3390/ijerph19052634/s1; Table S1: Calibration curve data for several parameters evaluated in this study; Table S2: Summary of collection days for the analysed samples; Table S3: VMS calibration curve data correspondent to the water samples; Table S4: VMS calibration curve data correspondent to the biomass samples; Figure S1: Time-course evolution of pH (a) and temperature (b) in each experiment; Figure S2: Time-course evolution of OD_680 nm_ in each experiment.

## Appendix A

Table A1 shows the colour and turbidity measurements of the cell-free culture media at the beginning and end of each experiment.

**Table A1:** Colour and turbidity measurements of the cell-free culture media at the beginning and end of each experiment.

Parameter	Experiment	Time	
		t_0_	t_f_
Colour (HU)	C+	6.4 ± 0.5 *	10.5 ± 0.8 *
	C-PE	76 ± 8 ^a^	47 ± 10 ^b^
	PE	65 ± 6 ^a^	68 ± 3 ^a^
	C-SE	29 ± 7 ^a^	20 ± 3 ^a^
	SE	30 ± 1 ^a^	55 ± 7 ^b^
Turbidity (NTU)	C+	0.9 ± 0.3 ^a^	1.8 ± 0.9 ^a^
	C-PE	5.5 ± 0.4 ^a^	2.8 ± 0.4 ^b^
	PE	4.8 ± 0.8 ^a^	3.3 ± 0.5 ^b^
	C-SE	0.8 ± 0.2 ^a^	0.70 ± 0.09 ^a^
	SE	1.2 ± 0.3 ^a^	2.4 ± 0.9 ^a^

HU: Hazen units; NTU: nephelometric turbidity unit; t_0_: time correspondent to the beginning of the experiment; t_f_: time correspondent to the end of the experiment; * values below the method’s limit of quantification. Values are presented as the mean ± standard deviation obtained from two independent experiments; within the same row, average values sharing at least one common letter (^a^ and ^b^) are not statistically different (*p* > 0.05).

## Appendix B

Figure A1 presents the time-course evolution of D3, D4 and D6 concentrations for the C-PE and PE experiments.
