# Supplementary material for: Microalgal Cultures for the Bioremediation of Urban Wastewaters in the Presence of Siloxanes
Source: Int J Environ Res Public Health. 2022 Feb 24;19(5):2634. doi: 10.3390/ijerph19052634 (PMC8909507; doi:10.3390/ijerph19052634)
Supplement: Supplementary file 1 [file ijerph-19-02634-s001.zip › ijerph-1577040-supplementary.pdf]

Supplementary materials

Microalgal cultures for the bioremediation of urban wastewaters in the presence of siloxanes

E.M. Salgado, A.L. Gonçalves, F. Sánchez-Soberón, N. Ratola, J.C.M. Pires

LEPABE - Laboratory for Process Engineering, Environment, Biotechnology and Energy, Faculty of Engineering, University of Porto, Rua Dr. Roberto Frias, 4200-465 Porto, Portugal.

* Correspondence: jcpires@fe.up.pt; Tel.: +351-22-508-2262

Table S1 Calibration curves data for several parameters evaluated in this study

x	y	a ± s_a	b ± s_b	R^2	LOD	LOQ	n_s
Biomass concentration (g_{DW} L^{-1})	$OD_{680\ nm}$	2.50 ± 0.09	-2.40 ± 0.11	0.996	0.13	0.45	5
NO_3^- (mg L^{-1})	$Abs_{410\ nm}$	$1.19\times10^{-2} \pm 1.18\times10^{-4}$	$4.59\times10^{-2} \pm 3.31\times10^{-3}$	1.00	0.83	2.78	7
KH_2PO_4 (mg L^{-1})	$Abs_{820\ nm}$	$6.19\times10^{-2} \pm 7.92\times10^{-4}$	$-5.70\times10^{-3} \pm 2.88\times10^{-2}$	0.999	1.4	4.6	6
TP (mg L^{-1})	$Abs_{880\ nm}$	$6.06\times10^{-1} \pm 5.94\times10^{-3}$	$3.90\times10^{-3} \pm 1.67\times10^{-3}$	0.997	0.008	0.028	7
Colour (HU)	$Abs_{400\ nm}$	$9.28\times10^{-4} \pm 7.66\times10^{-6}$	$-2.23\times10^{-3} \pm 1.31\times10^{-3}$	0.999	4.2	14	13
COD- low range (mg O_2 L^{-1})	$Abs_{410\ nm}$	$2.76\times10^{-3} \pm 6.79\times10^{-5}$	$9.67\times10^{-3} \pm 3.25\times10^{-3}$	0.997	3.5	12	7
COD- high range (mg O_2 L^{-1})	$Abs_{410\ nm}$	$4.23\times10^{-4} \pm 6.05\times10^{-6}$	$2.33\times10^{-3} \pm 3.25\times10^{-3}$	0.999	23	77	7

a: slope of the calibration curve; Abs: absorbance; b: intercept; COD: chemical oxygen demand; DW: dry weight; HU: Hazen units; KH_2PO_4: monopotassium phosphate; LOD: limit of detection; LOQ: limit of quantification; NO^{3-}: nitrate; ns: number of standards; OD: optical density; R^2: coefficient of determination; s_a: standard deviation of the slope; s_b: standard deviation of the intercept; TP: total phosphorus.

The limits of detection (LOD) and quantification (LOQ) were determined according to Equations S1 and S2, in which s_b corresponds to the standard deviation of the intercept and *a* represents the slope of the calibration curve.

$$LOD = \frac{3 \times s_b}{a} \quad (S1)$$

$$LOQ = \frac{10 \times s_b}{a} \quad (S2)$$

Table S2 Summary of the sample collection days in each experiment

Parameter	Culture sample days			Sample volume per day (mL)
	C+	C-PE / PE	C-SE / SE	
Optical density	0, 1, 2, 3, 6, 7, 8, 9	0, 1, 2, 3, 6, 7, 8, 9	0, 1, 2, 3, 6, 7	5
Biomass dry weight	0, 2, 3, 6, 8, 9	0, 2, 3, 6, 8, 9	0, 2, 3, 6, 7	25
TN, DOC, TDC, DIC and COD	0, 1, 2, 3, 6, 8, 9	0, 1, 2, 3, 6, 8, 9	0, 7	50
PO_4-P and NO_3-N	0, 1, 2, 3, 6, 7, 8, 9	0, 1, 2, 3, 6, 7, 8, 9	0, 1, 2, 3, 6, 7	15
VMSs in water	0, 1, 2, 3, 6, 7, 8, 9	0, 1, 2, 3, 6, 7, 8, 9	0, 1, 2, 3, 6, 7	70
VMSs in gas	0, 9	0,9	0,7	1 000
Colour and turbidity	0, 9	0, 9	0, 7	25

COD: chemical oxygen demand; C+: positive control assay; C-PE: negative control assay with primary effluent; C-SE: negative control assay with secondary effluent; DIC: dissolved inorganic carbon; DOC: dissolved organic carbon; NO_3-N: nitrate-nitrogen; PE: assay with primary effluent; PO_4-P: phosphate-phosphorus; SE: assay with secondary effluent; TN: total nitrogen; TDC: total dissolved carbon; VMSs: volatile methylsiloxanes. For the negative controls, samples for the determination of biomass dry weight were not collected.

Table S3 VMSs calibration curve data correspondent to the water samples

x (µg L^{-1})	y	a ± s_a (L µg^{-1})	R^2	LOD (µg L^{-1})	LOQ (µg L^{-1})	n_s	Linearity range (µg L^{-1})
C_{L5}	A_{L5}/A_{IS}	4.61×10^{-3} ± 4.47×10^{-5}	0.999	0.0053	0.018	9	
C_{D3}	A_{D3}/A_{IS}	5.43×10^{-3} ± 6.06×10^{-5}	0.999	0.14	0.45	11	
C_{D4}	A_{D4}/A_{IS}	5.30×10^{-3} ± 8.58×10^{-5}	0.997	0.18	0.59	11	1-1500
C_{D5}	A_{D5}/A_{IS}	2.94×10^{-3} ± 1.12×10^{-5}	1.00	0.0059	0.020	9	
C_{D6}	A_{D6}/A_{IS}	3.48×10^{-3} ± 5.43×10^{-5}	0.998	0.11	0.36	11	

CVMS: volatile methylsiloxanes concentration; L5: dodecamethylpentasiloxane; D3: hexamethylcyclotrisiloxane; D4: octamethylcyclotetrasiloxane; D5: decamethylcyclopentasiloxane; D6: dodecamethylcyclohexasiloxane; a: slope of the calibration curve; s_a: standard deviation of the slope; R^2: coefficient of determination; LOD: limit of detection; LOQ: limit of quantification; ns: number of standards.

Table S4 VMSs calibration curve data correspondent to the biomass samples

x (µg L^{-1})	y	a ± s_a (L µg^{-1})	R^2	LOD (µg L^{-1})	LOQ (µg L^{-1})	n_s	Linearity range (µg L^{-1})
C_{L5}	A_{L5}/A_{IS}	2.10×10^{-3} ± 1.33×10^{-5}	0.999	0.0084	0.028	9	
C_{D3}	A_{D3}/A_{IS}	2.97×10^{-3} ± 4.76×10^{-5}	0.998	0.19	0.64	9	
C_{D4}	A_{D4}/A_{IS}	2.56×10^{-3} ± 3.65×10^{-5}	0.998	0.25	0.85	9	5-1500
C_{D5}	A_{D5}/A_{IS}	1.49×10^{-3} ± 2.45×10^{-5}	0.998	0.0042	0.014	9	
C_{D6}	A_{D6}/A_{IS}	1.73×10^{-3} ± 2.56×10^{-5}	0.998	0.13	0.43	9	

a: slope of the calibration curve; CVMS: volatile methylsiloxanes concentration; D3: hexamethylcyclotrisiloxane; D4: octamethylcyclotetrasiloxane; D5: decamethylcyclopentasiloxane; D6: dodecamethylcyclohexasiloxane; LOD: limit of detection; LOQ: limit of quantification; L5: dodecamethylpentasiloxane; ns: number of standards; R2: coefficient of determination; s_a: standard deviation of the slope.

The LOD and LOQ for each calibration curve correspondent to a specific VMS were determined according to Equations S3 and S4, in which C_{LSTD} corresponds to the lowest concentration standard in ng L^{-1} and SNR_{LSTD} represents the signal-to-noise ratio obtained by the GC-MS for that standard.

$$\mathrm{LOD}=\frac{3\times C_{LSTD}}{SNR_{LSTD}} \qquad \text{(S3)}$$

$$\mathrm{LOQ}=\frac{10\times C_{LSTD}}{SNR_{LSTD}} \qquad \text{(S4)}$$

The concentration of a certain VMS in a water sample, C_w (ng L^{-1}), was determined according to Equation S5, in which: (i) $\left(\frac{A_{VMS}}{A_{IS}}\right)_{ws}$ is the ratio between the peak area for the VMS and the peak area for the IS for the water sample; (ii) $\left(\frac{A_{VMS}}{A_{IS}}\right)_{LB}$ is the ratio between the peak area for the VMS and the peak area for the IS correspondent to the LB; (iii) a is the slope of the respective calibration curve in µg L^{-1}; (iv) V_{cs} is the concentrated sample volume (0.5 mL) and (v) V_s is the sample volume (30 mL).

$$C_w=\frac{\left(\frac{A_{VMS}}{A_{IS}}\right)_{ws}-\left(\frac{A_{VMS}}{A_{IS}}\right)_{LB}}{a}\times\frac{V_{cs}}{V_s}\times 10^3 \qquad \text{(S5)}$$

The estimated mass of a certain VMS in the cells-free culture medium at the beginning of each experiment, M_{w0} (ng), was calculated as represented in Equation S6, in which C_{w0} is the VMS

concentration of the samples collected at this time (ng L^{-1}) and V_0 corresponds to the initial culture volume (L). Equation S7 represents the determination of the estimated VMS mass at the end of each experiment, M_{wf} (ng), where C_{wf} corresponds to the VMS concentration of the samples collected at this time (ng L^{-1}) and $V_{samples}$ represents the total volume of the samples collected over the course of each experiment (L).

$$M_{w0} = C_{w0} \times V_{t0} \quad (S6)$$

$$M_{wf} = C_{wf} \times (V_0 - V_{samples}) \quad (S7)$$

The VMS concentration in lyophilised biomass samples, C_b (ng L^{-1}), was determined according to Equation S8, in which $\left(\frac{A_{VMS}}{A_{IS}}\right)_{bs}$ is the ratio between the peak area for the VMS and the peak area for the IS corresponding to the biomass sample. The mass of VMS per mass of lyophilised biomass samples, $C_{s/b}$ (ng g_{DW}^{-1}), was calculated as represented in Equation S9, where V_e is the extract volume (1 mL) and M_b is the amount of lyophilised biomass (g_{DW}) used in the extraction procedure for that sample. The estimated amount of VMS in biomass at the end of the experiment, in each culture, M_b (ng), was calculated according to Equation S10, where X_f corresponds to the final biomass concentration in g_{DW} L^{-1}.

$$C_b = \frac{\left(\frac{A_{VMS}}{A_{IS}}\right)_S - \left(\frac{A_{VMS}}{A_{IS}}\right)_{LB}}{a} \times 10^3 \quad (S8)$$

$$C_{s/b} = \frac{C_b \times V_e}{M_{lb}} \quad (S9)$$

$$M_b = C_{s/b} \times (V_0 - V_{samples}) \times X_f \quad (S10)$$

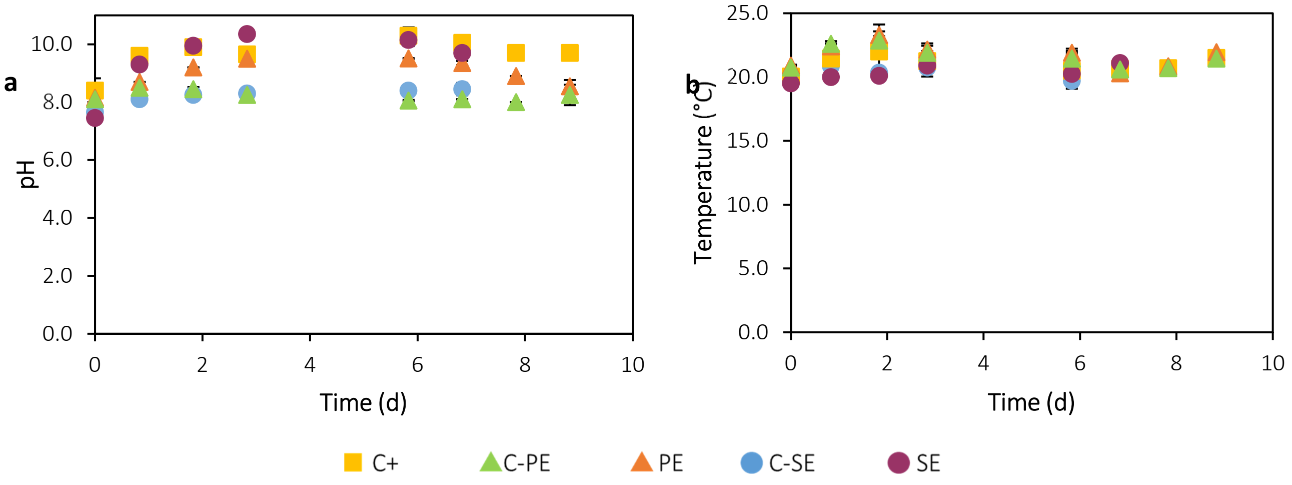

Figure S1 Time-course evolution of pH (a) and temperature (b) in each experiment. Error bars correspond to the standard deviation of the mean obtained from two independent experiments.

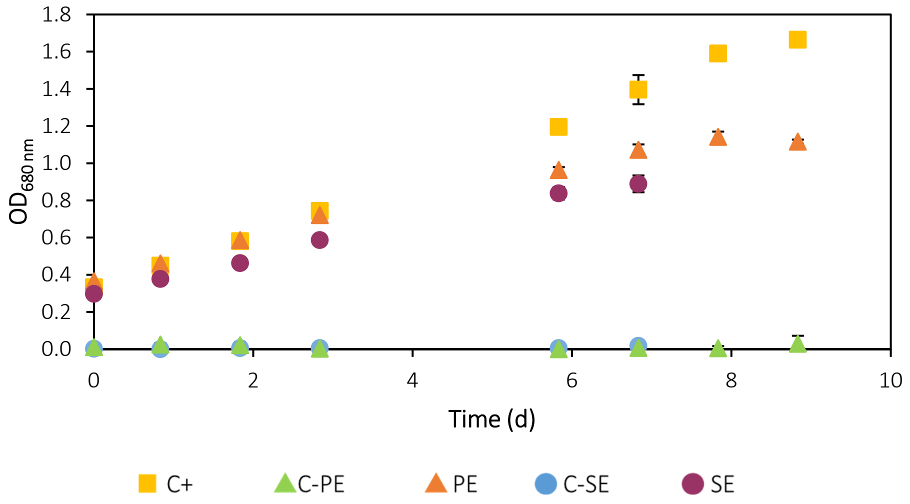

Figure S2 Time-course evolution of $OD_{680\,nm}$ in each experiment. Error bars correspond to the standard deviation of the mean obtained from two independent experiments.
